# The agreement between chronic diseases reported by patients and derived from administrative data in patients undergoing joint arthroplasty

**DOI:** 10.1186/s12874-019-0729-5

**Published:** 2019-04-24

**Authors:** Bélène Podmore, Andrew Hutchings, Sujith Konan, Jan van der Meulen

**Affiliations:** 1 Department of Health Services Research & Policy, London School of Hygiene & Tropical Medicine, UK, Clinical Effectiveness Unit, The Royal College of Surgeons of England, 15-17 Tavistock Place, London, WC1H 9SH UK; 2 Consultant Orthopaedic Surgeon, University College London Hospitals NHS Foundation Trust, London, UK

**Keywords:** Patient-report, Administrative data, Chronic diseases, Hip arthroplasty, Knee arthroplasty

## Abstract

**Background:**

This study examined the agreement between patient-reported chronic diseases and hospital administrative records in hip or knee arthroplasty patients in England.

**Methods:**

Survey data reported by 676,428 patients for the English Patient Reported Outcome Measures (PROMs) programme was linked to hospital administrative data. Sensitivity and specificity of 11 patient-reported chronic diseases were estimated with hospital administrative data as reference standard.

**Results:**

Specificity was high (> 90%) for all 11 chronic diseases. However, sensitivity varied by disease with the highest found for ‘diabetes’ (87.5%) and ‘high blood pressure’ (74.3%) and lowest for ‘kidney disease’ (18.8%) and ‘leg pain due to poor circulation’ (26.1%). Sensitivity was increased for diseases that were given as specific examples in the questionnaire (e.g. ‘parkinson’s disease’ (65.6%) and ‘multiple sclerosis’ (69.5%), compared to ‘diseases of the nervous system’ (20.9%)).

**Conclusions:**

Patients can give information about the presence of chronic diseases that is consistent with chronic diseases derived from hospital administrative data if the description in the patient questionnaire is precise and if the disease is familiar to most patients and has significant impact on their life. Such patient questionnaires need to be validated before they are used for research and service evaluation projects.

**Electronic supplementary material:**

The online version of this article (10.1186/s12874-019-0729-5) contains supplementary material, which is available to authorized users.

## Background

Patient surveys are often used in epidemiology to collect health data. However, the reliability and accuracy of patient-reported data, including patients’ own accounts of whether or not they have been diagnosed with a particular chronic disease, have been questioned [1]. Administrative data – hospital data collected for a range of administrative purposes including managing payments to the healthcare providers for every hospital admission and procedure – offer an alternative source of data [2].

To be able to record accurately chronic diseases is essential. Healthcare providers depend on accurate coding to be reimbursed for the care they provide especially when treating complex patients with multiple chronic diseases. In patients undergoing hip and knee arthroplasty the number of complex patients is likely to rise with more than 60% of patients for these operations reporting at least one comorbid chronic disease [3]. This number is expected to continue to rise as the number of people living with multiple chronic diseases is on the increase [4]. In addition, good quality coding is essential when looking at outcomes of hip and knee arthroplasty which may be affected by chronic diseases and analyses must therefore adjust for this effect.

Few studies have assessed the consistency of patient-reported chronic diseases with chronic diseases derived from administrative data [5–7]. The studies that did were predominantly cohort studies with relatively small sample sizes that reported single measures of agreement, such as the kappa statistic [1, 8]. A few larger scale studies investigated the agreement of a small number of patient-reported chronic diseases, with the most common being high blood pressure, stroke, heart disease and diabetes [5–7]. These studies found results for the agreement between patient-reported chronic diseases and hospital administrative data to vary significantly [1, 9–11].

We used the national Patient-Reported Outcome Measures (PROMs) programme of the English National Health Service (NHS), one of the largest collections of patient-reported data in the world, to assess the agreement of patient-reported chronic diseases against disease condition derived from hospital administrative data in patients undergoing hip or knee arthroplasty.

## Methods

### Study sample

The study sample of 676,428 patients was drawn from patient-reported data collected by the national PROMs programme in the English NHS [12]. All hospitals providing elective hip or knee arthroplasty funded by the English NHS are required to participate and patients are asked to complete pre-operative and post-operative questionnaires about their hip or knee condition and general health.

The data sample comprised completed pre-operative questionnaires linked with routinely collected administrative hospital data, Hospital Episode Statistics (HES) data, on all patient who had a hip or knee arthroplasty carried out in the English NHS between April 2009 and March 2016. The HES database contains a record of every inpatient hospital admission in the English NHS and is used primarily for reimbursement purposes [13]. A linked pre-operative PROMs questionnaire and HES record is available for 71% of eligible hip and knee arthroplasties [12].

We created a dataset comprising one unique linked patient-reported record for each individual patient. Duplicate records were excluded if more than one pre-operative questionnaire was linked to a procedure or more than one procedure in HES was linked to the same questionnaire. The first linked HES record for each patient was included but linked records for any subsequent procedures were excluded. Patients were also excluded if they reported seven or more comorbidities in the preoperative PROMs questionnaire due to the concerns about the validity of the responses. Patients appeared to report the absence rather than the presence of a chronic disease.

### Chronic disease according to the PROMs programme

In the PROMs pre-operative questionnaire patients were asked: ‘Have you ever been told by a doctor that you have any of the following conditions? Heart disease (for example angina, heart attack or heart failure), high blood pressure, problems caused by stroke, leg pain when walking due to poor circulation, lung disease (for example asthma, chronic bronchitis or emphysema), diabetes, kidney disease, diseases of the nervous system (for example, Parkinson’s disease or multiple sclerosis, liver disease, cancer (within the last 5 years), depression, [or] arthritis’. ‘Arthritis’ was excluded from our analyses because it is the primary a reason for hip or knee arthroplasty (81% patients reported having arthritis).

### Chronic disease according to administrative data

The 11 patient-reported chronic diseases were identified within HES data using International Classification of Disease (ICD-10) codes from the corresponding linked HES record of the hip or knee arthroplasty and from HES records of any other hospital admission within the previous 12 months or five years. Each HES record includes up to 20 ICD-10 diagnosis codes.

The initial set of ICD-10 codes for each of the 11 chronic diseases was derived from three chronic disease indices that have been used to identify chronic diseases in administrative data: The Royal College of Surgeons of England Charlson Comorbidity Index (RCS CCI) [14], the Quan Charlson Comorbidity Index (Quan CCI) [15] and the Elixhauser Comorbidity index [16]. The RCS CCI was chosen because it was designed to predict outcomes in surgical patients and has been validated for total hip arthroplasty using English HES data [14]. The Quan CCI is an adaptation of the Deyo CCI [15], and was chosen because it uses ICD-10 coding and is similar to other CCI adaptations in predicting both short-term and long-term mortality [17]. The Elixhauser Comorbidity Index was chosen because there is evidence that it may predict mortality better than other adaptations of the CCI [18].

The set of ICD-10 codes derived from the three chosen comorbidity indices were then mapped to the 11 diseases included in the PROMs questionnaire (see Additional file 1). A further 16 ICD-10 codes were added to the chronic disease mapping through the process of ‘backward coding’. ‘Backward coding’ involved reviewing linked HES records of hospital admissions in patients who had reported a chronic disease but who had no mapped records (ICD-10 codes) of the chronic disease in their HES records. First, relevant ICD-10 chapters were identified for each of the 11 chronic diseases. The most common (> 1% of patients reporting the chronic disease) and clinically relevant codes at the ICD-10 three-character category level were then identified. Second, the codes identified at the ICD-10 three-character level were further investigated at the ICD-10 four-character subcategory level. The prevalence of each four-character code in the administrative data was compared between patients who had and those who had not reported a specific chronic disease. The four-character code was added to the mapped ICD-10 codes if the proportion of patients reporting presence of a chronic disease was at least twice that in patients not reporting the chronic disease. For the main analyses, this final set of codes was used to determine the presence of chronic disease according to administrative data from the corresponding linked hospital record and from records of admissions within the previous 12 months or five years.

### Statistical analysis

The patient-reported chronic diseases at the point of surgery were compared with recorded diagnoses in the corresponding administrative record of the linked hospital admission and the records of previous admissions in two ways. First, agreement between patient-reported and administrative records was evaluated using sensitivity and specificity with administrative data as the reference standard. Second, we calculated the kappa statistic as an alternative measure of the agreement between patient-reported and administrative data for each condition. The kappa statistic is an agreement measure that takes into account chance agreement. A value of one indicates perfect agreement and a value of zero indicates no agreement above that expected by chance. Kappa values are often categorised in the following way: < 0.40 ‘poor agreement’, 0.40–0.60 ‘moderate agreement’, 0.61–0.80 ‘substantial agreement’, and 0.81–1.00 ‘near perfect agreement’ [19].

The sensitivity of patient-reported chronic disease was also explored further at the chronic disease subcategory level derived from administrative data. We grouped the set of ICD-10 codes for each of the 11 comorbid conditions according to clinically relevant subcategories (see Additional file 1). ICD-10 codes were grouped according to whether they reflected a cause (e.g. subarachnoid haemorrhage), a manifestation (e.g. asthma), or a consequences of disease (e.g. renal failure). For each comorbid condition ICD-10 codes that did not fit into any these grouping, the codes were put into an ‘other’ group. The sensitivity of the patient-reported chronic diseases compared to these chronic disease subcategories derived from administrative data was presented in a forest plot.

### Sensitivity analysis

The impact of the length of the look-back period on the performance of the combined chronic disease measure in administrative data was also investigated [20–22]. Some chronic diseases such as ‘heart disease’ are diseases that can fluctuate and others, such as ‘stroke’ are single events. For that type of chronic diseases, a longer look-back ensures that records of chronic diseases coded in admissions that occurred further in the past are also captured. In the PROMs questionnaire, patients were asked to recall cancer within the last five years which is another reason to use a 5-year look-back period as an alternative to the one-year look-back period.

## Results

### Study sample

**Fig. 1:**
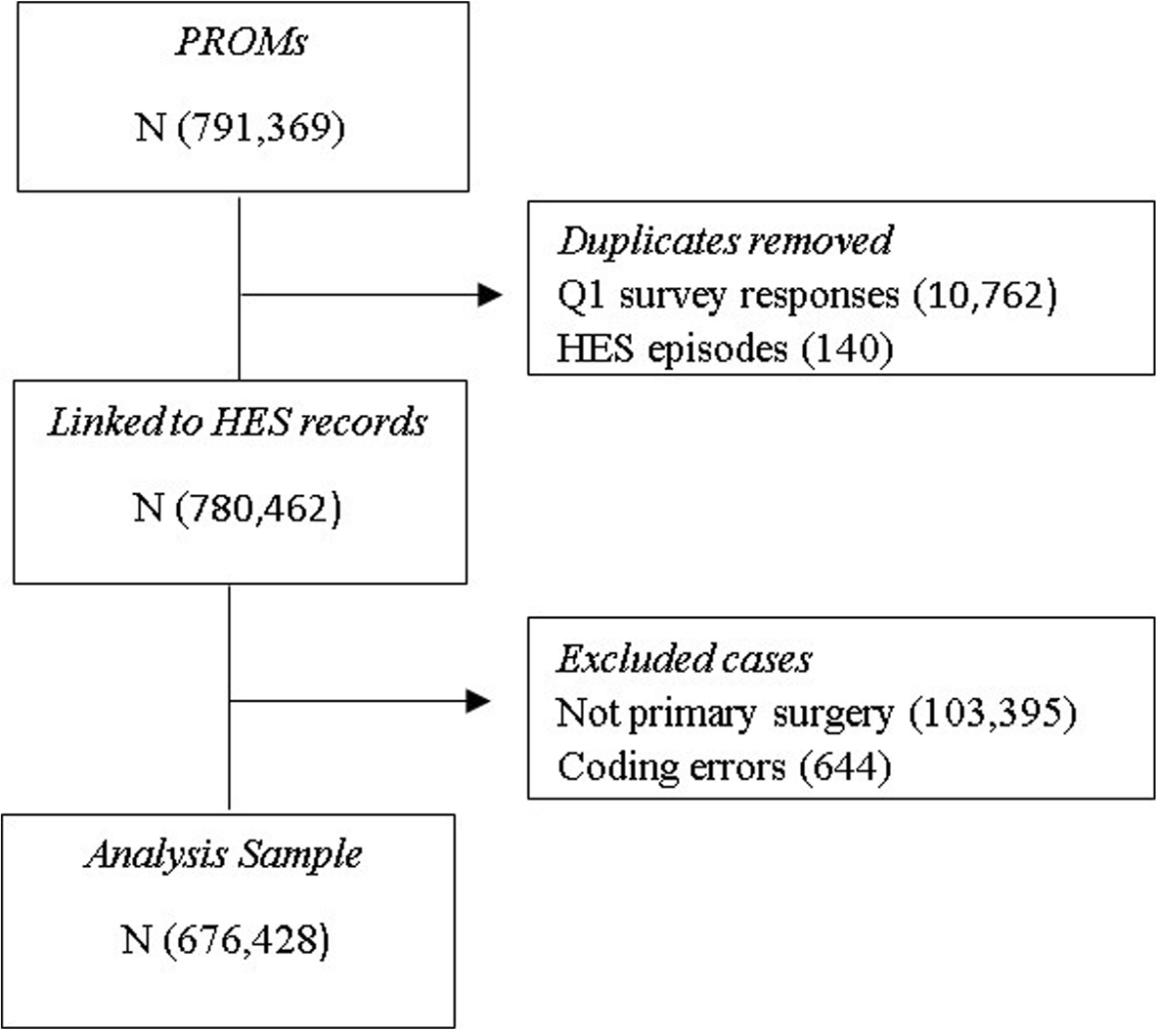
Flow chart

Agreement between chronic disease measures reported by patients and derived from administrative data was examined in 676,428 patients who underwent a hip or knee arthroplasty between 2009 and 2016 in the English NHS and who participated in the PROMs programme from a total 791,369 linked records. Records were excluded for the following reasons: duplicate pre-operative questionnaires (10,762), duplicate HES procedures (140), subsequent procedures for patients included in the analyses (103,395), and patients reporting seven or more chronic diseases on their pre-operative questionnaire (644) (see Fig. 1). 50.6% of the patients underwent knee arthroplasty. The average age of the population was 68 years (18–105). The majority of the patients had a white ethnic background (86.3%) and 58.0% of the study cohort were female (see Table 1). Patients living in the most socioeconomically deprived areas were slightly under-represented in the sample: those in the bottom two deprivation groups based on quintiles made up only 34.5% of patients undergoing a primary hip or knee arthroplasty whereas 40% is expected given that the quintiles reflect the national distribution.

**Table 1:** Characteristic of PROMs study population (*N* = 676,428)

	Number (%)
Age (mean, range)	69 (18–105)
Gender	
Male	283,892 (42.0)
Female	392,107 (58.0)
Missing, not stated	429 (0.06)
Socioeconomic status by quintile group	
1 (least deprived)	151,850 (22.5)
2	159,353 (23.6)
3	125,160 (18.5)
4	118,487 (17.5)
5 (most deprived)	114,691 (17.0)
Missing, not stated	6887 (1.02)
Ethnicity	
White or White British	583,674 (86.3)
Mixed background	1469 (0.22)
Asian or Asian British	12,126 (1.79)
Black or Black British	5377 (0.79)
Chinese or other ethnic	2991 (0.44)
Missing, not stated	70,791 (10.5)

### Agreement between patient-reported chronic disease and administrative data

Sensitivity, specificity, and the kappa statistic for patient-reported chronic disease against chronic diseases derived from administrative data using a 1-year look-back are reported in Table 1. Patient-reported chronic diseases had high specificity (ranging between 90.3% for ‘high blood pressure’ and 99.7% for ‘disease of the nervous system’ and ‘liver disease’), but sensitivity varied (ranging from 18.8% for ‘kidney disease’ to 87.5% for ‘diabetes’).

According to the kappa statistic, there was ‘substantial agreement’ between patient-reported and administrative results for ‘high blood pressure’ (κ =0.65) and ‘almost perfect agreement’ for ‘diabetes’ (κ =0.88) (see Table 2). There was ‘moderate agreement’ for ‘heart disease’ (κ =0.54) and ‘lung disease’ (κ =0.55). In contrast, there was ‘poor agreement’ for ‘stroke’, ‘liver disease’, ‘leg pain due to poor circulation’, ‘kidney disease’ and ‘depression’. Agreement between patient-reported chronic diseases and chronic disease subcategories derived from administrative data.

**Table 2:** Sensitivity and specificity of patient-reported chronic diseases relative to chronic diseases derived from administrative data (1-year look-back) (N = 676,428)

Chronic disease	Patient-reported *n* (%)	Administrative data n (%)	Prevalence in either patient-reported or administrative data, *n* (%)				Sensitivity (%) (95% CI)	Specificity (%) (95% CI)	Kappa (κ) (95% CI)
			Both	Administrative only	Patient-reported only	Neither			
Heart disease	67,425	122,219	56,736	65,460	10,689	543,543	46. 4	98.1	0.54
	(9.97)	(18.1)	(8.39)	(9.68)	(1.58)	(80.4)	(46.2, 46.7)	(98.0, 98.1)	(0.54, 0.54)
High blood pressure	282,785	335,958	249,608	86,350	33,177	307,293	74.3	90.3	0.65
	(41.8)	(49.7)	(36.9)	(12.8)	(4.90)	(45.4)	(74.1, 74.4)	(90.2, 90.4)	(0.64, 0.65)
Stroke	11,126	7348	2367	4981	8759	660,321	32.2	98.7	0.25
	(1.64)	(1.09)	(0.35)	(0.74)	(1.29)	(97.6)	(31.1, 33.3)	(98.7, 98.7)	(0.24, 0.25)
Leg pain due to poor circulation	48,298	10,917	2855	8063	45,444	620,067	26.1	93.2	0.07
	(7.14)	(1.61)	(0.42)	(1.19)	(6.72)	(91.7)	(25.3, 27.0)	(93.1, 93.2)	(0.07, 0.07)
Lung disease	55,717	100,260	46,876	53,384	8841	567,327	46.8	98.5	0.55
	(8.24)	(14.8)	(6.93)	(7.89)	(1.31)	(83.9)	(46.4, 47.1)	(98.4, 98.5)	(0.55, 0.56)
Diabetes	75,998	78,816	68,952	9864	7046	590,566	87.5	98.8	0.88
	(11.2)	(11.7)	(10.2)	(1.46)	(1.04)	(87.3)	(87.3, 87.7)	(98.8, 98.8)	(0.87, 0.88)
Kidney disease	12, 435	36,823	6910	29,913	5542	634,080	18.8	99.1	0.26
	(1.84)	(5.44)	(1.02)	(4.42)	(0.82)	(93.7)	(18.4, 19.2)	(99.1, 99.2)	(0.26, 0.26)
Diseases of the nervous system	5840	19,550	4092	15,458	1748	655,130	20.9	99.7	0.31
	(0.86)	(2.89)	(0.60)	(2.29)	(0.26)	(96.9)	(20.4, 21.5)	(99.7, 99.7)	(0.31, 0.32)
Liver disease	3585	4120	1412	2708	2173	670,135	34.3	99.7	0.36
	(0.53)	(0.61)	(0.21)	(0.40)	(0.32)	(99.1)	(32.8, 35.7)	(99.7, 99.7)	(0.36, 0.37)
Cancer	32,384	12,710	8740	3970	23,644	640,074	68.8	96.4	0.37
	(4.79)	(1.88)	(1.29)	(0.59)	(3.50)	(94.6)	(68.0, 69.6)	(96.4, 96.5)	(0.37, 0.37)
Depression	61,589	29,923	18,263	11,660	43,326	603,179	61.0	93.3	0.36
	(9.11)	(4.42)	(2.70)	(1.72)	(6.41)	(89.2)	(60.5 61.6)	(93.2, 93.4)	(0.36, 0.36)

**Fig. 2:**
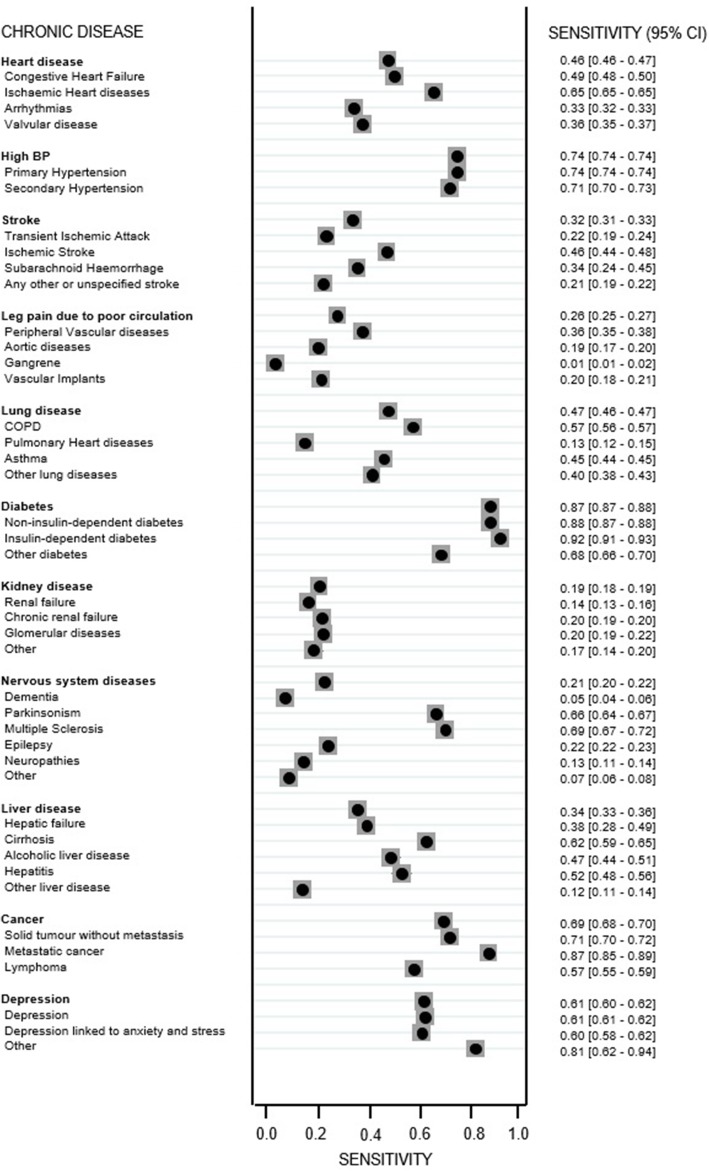
Forest plot of sensitivity by chronic disease subcategories derived from administrative data (95% CI)

Further investigation comparing patient-reported chronic disease against chronic disease subcategories derived from administrative data demonstrated that the sensitivity varied if the patient-reported results were compared against subcategories defined according to administrative data (see Fig. 2). Sensitivity ranged from 1.3% for patient-reported ‘leg pain due to poor circulation’ compared against ‘gangrene’ according to administrative data to 91.6% for patient-reported ‘diabetes’ compared against ‘insulin-dependent diabetes’ according to administrative data.

The sensitivity was considerably higher in subgroups of chronic diseases where specific examples of the chronic diseases were given as examples in the questionnaire used for the PROMs survey in the PROMs survey. For example, we saw that the sensitivity of ‘diseases of the nervous system (for example Parkinson’s disease or multiple sclerosis)’ was much higher in subgroups of patients who had these two specific diseases quotes as examples in their administrative data (65.6 and 69.5%, respectively) than in entire group of patients who had the generic term ‘diseases of the nervous system’ in the administrative data (20.9%). We saw a similar effect for the examples given in ‘heart disease (for example angina, heart attack or heart failure)’. The sensitivity in the subgroup of patients with the specific term ischemic heart disease in the administrative data was significantly higher (64.9%) than in all patients who had the generic term ‘heart disease’ according to administrative data (46.4%).

### Impact of length of look-back period on agreement

The impact of the length of look-back period on the chronic diseases derived from administrative data was investigated. Increasing the look-back period for identifying chronic diseases in administrative data from 12 months to five years had little impact on the sensitivity, specificity and kappa statistic (see Table 3). As expected, sensitivity decreased and specificity increased. The biggest change was the increase of the kappa statistic for ‘cancer’ from 0.37 with a 12-month look-back period to 0.69 with a 5-year look-back period.

**Table 3:** Sensitivity and specificity of patient -reported chronic disease relative to chronic disease derived from administrative data using a 5-year look-back period

Chronic disease	Prevalence *n* (%)	Sensitivity (%) (95% CI)	Specificity (%) (95% CI)	Kappa (κ) (95% CI)
Heart disease	141,457 (20.9)	43.0	98.8	0.52
		(42.7, 43.2)	(98.7, 98.8)	(0.52, 0.52)
High blood pressure	358,699 (53.0)	72.3	92.7	0.64
		(72.2, 72.5)	(92.6, 92.7)	(0.64,0.65)
Stroke	15,783 (2.33)	30.3	99.0	0.34
		(29.6, 31.1)	(99.0, 99.1)	(0.34. 0.34)
Leg pain due to poor circulation	17,728 (2.62)	24.1	93.3	0.10
		(23.5, 24.7)	(93.3, 93.4)	(0.10, 0.10)
Lung disease	112,774 (16.7)	43.6	98.8	0.53
		(43.3, 43.9)	(98.8, 98.9)	(0.53, 0.53)
Diabetes	82,384 (12.2)	85.6	99.1	0.88
		(85.3, 85.8)	(99.0, 99.1)	(0.88,0.88)
Kidney disease	45,172 (6.68)	17.1	99.3	0.25
		(16.8, 17.5)	(99.2, 99.3)	(0.25,0.25)
Diseases of the nervous system	24,727 (3.66)	17.4	99.8	0.27
		(17.0, 17.9)	(99.8, 99.8)	(0.27,0.27)
Liver disease	7173 (1.06)	24.6	99.7	0.32
		(23.6, 25,6)	(99.7, 99.7)	(0.32,0.32)
Cancer	31,649 (4.68)	71.2	98.5	0.69
		(70.7, 71.7)	(98.4, 98.5)	(0.69,0.69)
Depression	38,503 (5.69)	58.4	93.9	0.41
		(57.9, 58.9)	(93.8, 93.9)	(0.41,0.41)

## Discussion

In this large study of patients undergoing hip or knee arthroplasty we determined that for 11 patient-reported chronic diseases specificity was high but sensitivity varied greatly when the patient-reported results were compared to administrative data. Specifically, sensitivity was highest for ‘diabetes’ and ‘high blood pressure’ and lowest for ‘leg pain due to poor circulation’ and ‘stroke’. The variation in sensitivity also differed further when the patient-reported chronic diseases were compared against chronic diseases subcategories derived from administrative data. Sensitivity is high if the description of the chronic disease in the patient questionnaire is precise and uses language familiar to most patients, if it requires daily treatment or drug administration for the patient, or the chronic diseases has a significant impact on patient’s lives.

Sensitivity was high for comorbid conditions that describe a specific disease diagnosis (in terms of a cause, manifestation, or disease consequence) rather than a collection of symptoms. This might explain why ‘diabetes’ had higher sensitivity than ‘leg pain due to poor circulation’ and ‘problems caused by stroke’. Similarly, when looking at disease subcategories, sensitivity was higher when specific examples of chronic diseases were given in the PROMS questionnaire survey rather than the generic category for the chronic disease. This demonstrated that if a disease has a spectrum of severity, subcategories may be more useful categories to use to ask patients about the presence of any chronic diseases.

It is important to note that administrative hospital data, HES, is not a perfect reference standard. This may explain why the specificity, while generally high for all chronic diseases, varied by up to 10%. Certain chronic diseases may not be fully recorded in administrative data because they may not be severe enough to significantly alter the treatment a patient receives in hospital or influence the hospital’s resource use related to a patient’s care. Further coding errors in hospital administrative data can also occur as coding is often undertaken by administrative staff who depend on medical notes so any errors in the notes can lead to chronic diseases not being captured. This is likely to lead to an underestimation of the agreement between patient-reported chronic diseases and chronic diseases derived from administrative hospital data. On the other hand, conditions that are single events in time such as stroke and ischemic heart diseases may not be recorded in administrative data due to a limited look-back period [23–25]. Nevertheless, when we increased the look-back period from 12 months to five years there appeared to be little or no impact on sensitivity of patient-reported chronic disease relative to administrative data. An increase of the duration of the look-back period to five years did improve the agreement for cancer but this may just be a reflection of the PROMs question, which asked patients to report ever being diagnosed with cancer in the last five years.

A study comparing patient-reported chronic disease against chart review suggested that low agreement, especially low sensitivity, may be due to the description of the conditions in the patient questionnaire, for example if the wording is based more on symptoms (‘leg pain due to poor circulation’) than disease (‘diabetes) or if the disease has stable or only a few symptoms (e.g. ‘kidney disease’) [26]. Similarly, previous studies found that conditions requiring ongoing management such as diabetes or hypertension had highest agreements in comparison to poorly defined diseases such as stroke or congestive heart failure [5–7, 27].

With respect to the impact of the length of the look-back period, other studies had similar findings to ours in that they found limited benefits in increasing the look-back period beyond one year [6, 27].

These findings provide support for the use of patient-reported data to identify patients with chronic diseases if administrative data are unavailable. The questionnaire should however be validated beforehand with patients to ensure clarity, comprehension and ease of completion. This is especially important to improve the capture of less common and more complex chronic diseases such as kidney disease or diseases of the nervous system.

There are several limitations to this study. As is the case for any cohort study the generalisability of our conclusions are limited by the characteristics of our population and the quality of the data. The PROMs questionnaires were completed by patients who underwent hip or knee arthroplasty and as a consequence, these patients were likely to have fewer and less severe chronic diseases than a population of older patients with arthritis because more severe cases are less likely to be eligible for surgery [28]. The agreement measures used to compare different data sources also have known limitations. Agreement measures such as the Kappa statistic have been reported to be influenced by the prevalence of the diseases [29]. Previous studies on the validity of administrative data, have recommended the use of a minimum of four statistical measures to help mitigate these limitations [30, 31]. As a result, the prevalence, the raw frequency counts, sensitivity, specificity and the Kappa statistic were all reported. Disease status is often also not clear-cut and the recording in hospital administrative data – our reference standard – will often be based on a ‘cut-off point’ with most misclassification occurring in those patients with a true disease status close to the cut-off point. The combination of a relatively low prevalence and mild severity may therefore partly explain our finding of relatively low sensitivities and high specificities [32].

## Conclusions

This study indicates that patients can give information about the presence of chronic diseases that is consistent with chronic diseases derived from hospital administrative data. The sensitivity and specificity of patient-reported chronic disease can be high if the description in the patient questionnaire is precise and familiar to most patients and if the conditions have a specific impact on the patients’ lives. These findings may guide the development of questionnaires that can be used to ask patients whether or not they have particular chronic diseases.

## Additional file

Additional file 1: Mapping of chronic diseases and chronic disease subcategories by Comorbidity Index. (DOCX 38 kb)

## Abbreviations

CCI: Charlson Comorbidity Index
HES: Hospital Episodes Statistic
ICD-10: International Classification of Disease, Version 10
NHS: National Health Service
PROMs: Patient-Reported Outcome Measures
RCS: The Royal College of Surgeons of England
