# Supplementary material for: The agreement between chronic diseases reported by patients and derived from administrative data in patients undergoing joint arthroplasty
Source: BMC Med Res Methodol. 2019 Apr 24;19:87. doi: 10.1186/s12874-019-0729-5 (PMC6480886; doi:10.1186/s12874-019-0729-5)
Supplement: Supplementary file 1 — Mapping of chronic diseases and chronic disease subcategories by Comorbidity Index. (DOCX 38 kb) [file 12874_2019_729_MOESM1_ESM.docx]

# Additional file 1

| **PROMs chronic disease category** | **Chronic disease Subcategories** | **ICD-10 Code** | **ICD-10 definition** | **RCS CI** | **Quan CCI** | **Elixhauser*** | **Backward coding** |
| --- | --- | --- | --- | --- | --- | --- | --- |
| Cancer | Lymphoma | C81.x-C85.x | Malignant neoplasms | x | x | x |  |
|  |  | C88 | Malignant neoplasms | x | x | x |  |
|  |  | C90.0 | Malignant neoplasms | x | x | x |  |
|  |  | C90.2 | Malignant neoplasms | x | x | x |  |
|  |  | C90-C97 | Malignant neoplasms | x | x |  |  |
|  |  | C96.x | Malignant neoplasms | x | x | x |  |
|  | Metastatic cancer | C77–C80 | Malignant neoplasms | x | x | x |  |
|  | Solid Tumour without metastasis | C00.x-C26.x | Malignant neoplasms | x | x | x |  |
|  |  | C30.x-C34.x | Malignant neoplasms | x | x | x |  |
|  |  | C37.x-C41.x | Malignant neoplasms | x | x | x |  |
|  |  | C43 | Malignant neoplasms | x | x | x |  |
|  |  | C45.x-C58.x | Malignant neoplasms | x | x | x |  |
|  |  | C60.x-C76.x | Malignant neoplasms | x | x | x |  |
|  |  | C97.x | Malignant neoplasms | x | x | x |  |
| Depression | Depression | F32.x | Depressive episode |  |  | x |  |
|  |  | F33 | Recurrent depressive disorder |  |  | x |  |
|  |  | F34.1 | Dysthymia |  |  | x |  |
|  | Depression linked to anxiety and stress | F41.2 | Mixed anxiety and depressive disorder |  |  | x |  |
|  |  | F43.2 | Adjustment disorders |  |  | x |  |
|  | Other depression | F20.4 | Post-schizophrenic depression |  |  | x |  |
|  |  | F31.3-F31.5 | Bipolar affective disorder, current episode mild or moderate depression, severe depression without psychotic symptoms, severe depression with psychotic symptoms |  |  | x |  |
| Diabetes | Insulin-dependent diabetes | E10 | Insulin-dependent diabetes mellitus | x |  |  |  |
|  | Non-insulin-dependent diabetes | E11 | Non-insulin-dependent diabetes mellitus | x | x | x |  |
|  | Other | E12 | Malnutrition-related diabetes mellitus | x | x | x |  |
|  |  | E13 | Other specified diabetes mellitus | x | x | x |  |
|  |  | E14 | Unspecified diabetes mellitus | x | x | x |  |
| Heart disease | Cardiac arrhythmias | I441-I443 | Atrioventricular block, second degree, Atrioventricular block, complete, Other and unspecified atrioventricular block |  |  | x |  |
|  |  | I456 | Pre-excitation syndrome |  |  | x |  |
|  |  | I459 | Conduction disorder, unspecified |  |  | x |  |
|  |  | I47-I49 | Paroxysmal tachycardia, Atrial fibrillation and flutter, Other cardiac arrhythmias |  |  | x |  |
|  |  | R00.1 | Bradycardia, unspecified |  |  | x |  |
|  |  | R00.8 | Other and unspecified abnormalities of heart beat |  |  | x |  |
|  |  | R000 | Tachycardia, unspecified |  |  | x |  |
|  |  | T82.1 | Mechanical complication of cardiac electronic device |  |  | x |  |
|  |  | Z45.0 | Adjustment and management of cardiac pacemaker |  |  | x |  |
|  |  | Z95.0 | Presence of cardiac pacemaker |  |  | x |  |
|  | Congestive heart failure | I09.9 | Rheumatic heart disease, unspecified |  | x | x |  |
|  |  | I11.0 | Hypertensive heart disease with (congestive) heart failure |  | x | x |  |
|  |  | I13.0 | Hypertensive heart and renal disease with (congestive) heart failure | x | x | x |  |
|  |  | I13.2 | Hypertensive heart and renal disease with both (congestive) heart failure and renal failure |  | x | x |  |
|  |  | I255 | Ischaemic cardiomyopathy | x | x | x |  |
|  |  | I42 | Cardiomyopathy | x |  |  |  |
|  |  | I42.0 | Dilated cardiomyopathy | x | x | x |  |
|  |  | I42.5-I42.9 | Other restrictive cardiomyopathy, Alcoholic cardiomyopathy, Cardiomyopathy due to drugs and other external agents, Other cardiomyopathies, Cardiomyopathy, unspecified | x | x | x |  |
|  |  | I42.6 | Alcoholic cardiomyopathy | x |  | x |  |
|  |  | I43 | Cardiomyopathy in diseases classified elsewhere | x | x | x |  |
|  |  | I50 | Heart failure | x | x | x |  |
|  |  | I517 | Cardiomegaly | x |  |  |  |
|  | Ischemic heart diseases | I21 | Acute myocardial infarction | x | x |  |  |
|  |  | I22 | Subsequent myocardial infarction | x | x |  |  |
|  |  | I23 | Certain current complications following acute myocardial infarction | x | x |  |  |
|  |  | I252 | Old myocardial infarction | x |  |  |  |
|  |  | I258 | Other forms of chronic ischaemic heart disease |  |  |  | x |
|  |  | I259 | Chronic ischaemic heart disease, unspecified |  |  |  | x |
|  |  | I20 | Angina Pectoris |  |  |  | x |
|  |  | I251 | Atherosclerotic heart disease |  |  |  | x |
|  | Valvular disease | Q23.O-Q23.3 | Congenital stenosis of aortic valve, Congenital insufficiency of aortic valve, Congenital mitral stenosis, Congenital mitral insufficiency |  | x | x |  |
|  |  | Z95.2 | Presence of prosthetic heart valve |  | x | x |  |
|  |  | Z95.4 | Presence of other heart-valve replacement |  | x | x |  |
|  |  | A52.0 | Cardiovascular syphilis |  | x | x |  |
|  |  | I05.x-I08.x | Rheumatic mitral valve diseases, Rheumatic aortic valve diseases, Rheumatic tricuspid valve diseases, Multiple valve diseases |  | x | x |  |
|  |  | I09.1 | Rheumatic diseases of endocardium, valve unspecified |  | x | x |  |
|  |  | I09.8 | Other specified rheumatic heart diseases |  | x | x |  |
|  |  | I34-I39 | Nonrheumatic mitral valve disorders, Nonrheumatic aortic valve disorders, Nonrheumatic tricuspid valve disorders, Pulmonary valve disorders, Endocarditis, valve unspecified, Endocarditis and heart valve disorders in diseases classified elsewhere |  | x | x |  |
| High BP | Primary hypertension | I10.x | Essential (primary) hypertension |  |  | x |  |
|  | Secondary hypertension | I11 | Hypertensive heart disease | x |  | x |  |
|  |  | I15 | Secondary hypertension |  |  | x |  |
| Kidney disease | Acute renal failure | N171* | Acute renal failure with acute cortical necrosis | x |  |  |  |
|  |  | N172* | Acute renal failure with medullary necrosis | x |  |  |  |
|  |  | N179* | Acute renal failure, unspecified |  |  |  | x |
|  | Chronic renal failure | I12.0 | Hypertensive renal disease with renal failure |  | x | x |  |
|  |  | I13.1 | Hypertensive heart and renal disease with renal failure |  | x | x |  |
|  |  | N18 | Chronic kidney disease | x | x | x |  |
|  |  | N19 | Unspecified kidney failure | x | x | x |  |
|  |  | Z49 | Care involving dialysis | x |  |  |  |
|  |  | Z49.0-Z49.2 | Preparatory care for dialysis, Extracorporeal dialysis, Other dialysis | x | x | x |  |
|  |  | Z94.0 | Kidney transplant status | x | x | x |  |
|  |  | Z99.2 | Dependence on renal dialysis | x | x | x |  |
|  |  | N19 | Unspecified Kidney failure |  |  |  | x |
|  | Glomerular diseases | N01 | Rapidly progressive nephritic syndrome | x |  |  |  |
|  |  | N03 | Chronic nephritic syndrome | x |  |  |  |
|  |  | N03.2–N03.7 | Rapidly progressive nephritic syndrome | x | x |  |  |
|  |  | N05 | Unspecified nephritic syndrome | x |  |  |  |
|  |  | N05.2–N05.7 | Unspecified nephritic syndrome Diffuse membranous glomerulonephritis , Diffuse mesangial proliferative glomerulonephritis, Diffuse endocapillary proliferative glomerulonephritis, Diffuse mesangiocapillary glomerulonephritis | x | x |  |  |
|  |  | N07 | Hereditary nephropathy, not elsewhere classified | x |  |  |  |
|  |  | N08 | Glomerular disorders in diseases classified elsewhere | x |  |  |  |
|  | Other renal disease | N25 | Disorders resulting from impaired renal tubular function | x |  |  |  |
|  |  | N289 | Disorder of kidney and ureter, unspecified |  |  |  | x |
|  | Secondary hypertension | I12 | Hypertensive renal disease | x |  | x |  |
|  |  | I13 | Hypertensive heart and renal disease | x |  | x |  |
| Leg pain due to poor circulation | Aortic diseases | I70 | Atherosclerosis | x | x | x |  |
|  |  | I71 | Aortic aneurysm and dissection | x | x | x |  |
|  |  | I72 | Other aneurysm and dissection | x |  |  |  |
|  |  | I790 | Aneurysm of aorta in diseases classified elsewhere |  | x | x |  |
|  | Gangrene | R02 | Gangrene, not elsewhere classified | x |  |  |  |
|  | Peripheral vascular diseases | I73 | Other peripheral vascular diseases |  |  |  |  |
|  |  | I731 | Thromboangitis obliterans [Buerger] | x | x | x |  |
|  |  | I738 | Other specified peripheral vascular diseases | x | x | x |  |
|  |  | I739 | Peripheral vascular disease, unspecified | x | x | x |  |
|  |  | I770 | Arteriovenous fistula, acquired | x |  |  |  |
|  |  | I771 | Stricture of artery | x | x | x |  |
|  |  | I792 | Peripheral angiopathy in diseases classified elsewhere |  | x | x |  |
|  | Vascular implants | Z95.8 | Presence of other cardiac and vascular implants and grafts | x | x | x |  |
|  |  | Z95.9 | Presence of cardiac and vascular implant and graft, unspecified | x | x | x |  |
| Liver disease | Alcoholic liver disease | K70 | Alcoholic liver disease | x |  | x |  |
|  |  | K70.0 | Alcoholic fatty liver | x | x | x |  |
|  |  | K70.1 | Alcoholic hepatitis | x | x |  |  |
|  |  | K70.2 | Alcoholic fibrosis and sclerosis of liver | x | x |  |  |
|  |  | K70.3 | Alcoholic cirrhosis of liver | x | x | x |  |
|  |  | K70.4 | Alcoholic hepatic failure | x | x |  |  |
|  |  | K70.9 | Alcoholic liver disease, unspecified | x | x | x |  |
|  | Cirrhosis | I85.9 | Oesophageal varices without bleeding | x | x | x |  |
|  |  | I85.0 | Oesophageal varices with bleeding | x | x | x |  |
|  |  | I86.4 | Gastric varices | x | x | x |  |
|  |  | I98.2 | Oesophageal varices without bleeding in diseases classified elsewhere | x | x | x |  |
|  |  | K71.7 | Toxic liver disease with fibrosis and cirrhosis of liver | x | x | x |  |
|  |  | K74 | Fibrosis and cirrhosis of liver |  | x | x |  |
|  | Hepatic failure | K71.1 | Toxic liver disease with hepatic necrosis | x | x | x |  |
|  |  | K72.0* | Acute hepatic failure |  |  |  |  |
|  |  | K72.1 | Chronic hepatic failure | x | x | x |  |
|  |  | K72.9 | Hepatic failure, unspecified | x | x | x |  |
|  |  | K72.x | Hepatic failure, not elsewhere classified |  |  | x |  |
|  | Hepatitis | B18 | Chronic viral hepatitis | x | x | x |  |
|  |  | K71.3-K71.6 | Toxic liver disease with chronic persistent hepatitis, chronic lobular hepatitis, chronic active hepatitis | x | x | x |  |
|  |  | K73 | Chronic hepatitis, not elsewhere classified |  | x | x |  |
|  |  | K754 | Autoimmune hepatitis |  |  |  | x |
|  | Other | K76 | Other diseases of liver | x |  |  |  |
|  |  | R162 | Hepatomegaly with splenomegaly, not elsewhere classified | x |  |  |  |
|  |  | Z94.4 | Liver transplant status | x | x | x |  |
| Lung disease | Asthma | J45-46 | Asthma | x | x | x |  |
|  | COPD | J40-42 | Bronchitis | x | x | x |  |
|  |  | J43 | Emphysema | x | x | x |  |
|  |  | J44 | COPD | x | x | x |  |
|  | Pulmonary heart diseases | I26 | Pulmonary embolism | x |  | x |  |
|  |  | I278 | Other specified pulmonary heart diseases | x | x | x |  |
|  |  | I279 | Pulmonary heart disease, unspecified | x | x | x |  |
|  | Other lung disease (e.g. due to external agents) | J47 | Bronchiectasis |  |  |  |  |
|  |  | J60-J67 | Lung diseases due to external agents | x | x | x |  |
|  |  | J684 | Chronic respiratory conditions due to chemicals, gases, fumes and vapours | x | x | x |  |
|  |  | J701 | Chronic and other pulmonary manifestations due to radiation | x | x | x |  |
|  |  | J703 | Chronic drug-induced interstitial lung disorders | x | x | x |  |
|  |  | J841 | Other interstitial pulmonary diseases with fibrosis |  |  |  | x |
|  |  | J920 | Pleural plaque with presence of asbestos |  |  |  | x |
| Nervous System | Dementia | F00.x–F03.x | Dementia in Alzheimer disease, Vascular dementia, Dementia in other diseases classified elsewhere, Unspecified dementia | x | x |  |  |
|  |  | F051 | Delirium superimposed on dementia | x | x |  |  |
|  |  | G30 | Alzheimer disease | x | x |  |  |
|  |  | G31 | Other degenerative diseases of nervous system, not elsewhere classified | x |  |  |  |
|  |  | G31.1 | Senile degeneration of brain, not elsewhere classified | x | x |  |  |
|  |  | G31.2 | Degeneration of nervous system due to alcohol | x |  | x |  |
|  |  | G31.8 | Other specified degenerative diseases of nervous system | x |  | x |  |
|  |  | G31.9 | Degenerative disease of nervous system, unspecified | x |  | x |  |
|  |  | G32.x | Other degenerative disorders of nervous system in diseases classified elsewhere |  |  | x |  |
|  | Demyelinating disease | G35 | Multiple sclerosis |  |  | x |  |
|  |  | G36 | Other acute disseminated demyelination |  |  | x |  |
|  |  | G37 | Other demyelinating diseases of central nervous system |  |  | x |  |
|  | Epilepsy | G40 | Epilepsy |  |  | x |  |
|  |  | G41 | Status epilepticus |  |  | x |  |
|  |  | R56 | Convulsions, not elsewhere classified |  |  | x |  |
|  | Neuropathies | G600 | Hereditary motor and sensory neuropathy |  |  |  | x |
|  |  | G629 | Polyneuropathy |  |  |  | x |
|  |  | G610 | Guillain-Barré syndrome |  |  |  | x |
|  |  | G618 | Other inflammatory polyneuropathies |  |  |  | x |
|  |  | G619 | Inflammatory polyneuropathy, unspecified |  |  |  | x |
|  | Parkinsonism | G20.x-G22.x | Parkinson disease, secondary parkinsonism, parkinsonism in diseases classified elsewhere |  |  | x |  |
|  |  | G25.4 | Drug-induced chorea |  |  | x |  |
|  |  | G25.5 | Other chorea |  |  | x |  |
|  |  | G249 | Dystonia, unspecified |  |  |  | x |
|  |  | G250 | Essential tremor |  |  |  | x |
|  | Other nervous system (e.g. paralysis, huntington's disease) | A810 | Creutzfeldt-Jakob disease | x |  |  |  |
|  |  | G04.1 | Tropical spastic paraplegia |  |  | x |  |
|  |  | G10.x-G13 | Huntingdon disease, Hereditary ataxia, spinal muscular atrophy and related syndromes, Systemic atrophies primarily affecting central nervous system in diseases classified elsewhere |  |  | x |  |
|  |  | G11.4 | Hereditary spastic paraplegia | x |  | x |  |
|  |  | G80.1 | Spastic diplegic cerebral palsy |  |  | x |  |
|  |  | G80.2 | Spastic hemiplegic cerebral palsy |  |  | x |  |
|  |  | G82.x | Paraplegia and tetraplegia | x |  | x |  |
|  |  | G83 | Other paralytic syndromes | x |  |  |  |
|  |  | G83.0-G83.4 | Diplegia of upper limbs, Monoplegia of lower limb, Monoplegia of upper limb, Monoplegia, unspecified, Cauda equina syndrome | x |  | x |  |
|  |  | G83.9 | Paralytic syndrome, unspecified |  |  | x |  |
|  |  | G93.1 | Anoxic brain damage, not elsewhere classified |  |  | x |  |
|  |  | G93.4 | Encephalopathy, unspecified |  |  | x |  |
|  |  | R47.0 | Dysphasia and aphasia |  |  | x |  |
|  |  | G700 | Myasthenia |  |  |  | x |
|  |  | G933 | Postviral fatigue syndrome |  |  |  | x |
| Stroke | Transient Ischemic Attack | G45 | Transient cerebral ischaemic attacks and related syndromes | x | x |  |  |
|  | Ischemic stroke | I69.2 | Sequelae of other nontraumatic intracranial haemorrhage | x | x |  |  |
|  |  | G46 | [Vascular syndromes of brain in cerebrovascular diseases (I60-I67+)](http://apps.who.int/classifications/icd10/browse/2010/en#/I60) | x | x |  |  |
|  |  | H34.0 | Transient retinal artery occlusion |  | x |  |  |
|  |  | I61 | Intracerebral haemorrhage | x | x |  |  |
|  |  | I62 | Other nontraumatic intracranial haemorrhage | x | x |  |  |
|  |  | I63 | Cerebral infarction | x | x |  |  |
|  |  | I64 | Stroke, not specified as haemorrhage or infarction | x | x |  |  |
|  |  | I65 | Occlusion and stenosis of precerebral arteries, not resulting in cerebral infarction | x | x |  |  |
|  |  | I66 | Occlusion and stenosis of cerebral arteries, not resulting in cerebral infarction | x | x |  |  |
|  |  | I69.1 | Sequalae of intracerebral haemorrhage | x | x |  |  |
|  |  | I69.3 | Sequelae of cerebral infarction | x | x |  |  |
|  |  | I69.4 | Sequelae of stroke, not specified as haemorrhage or infarction | x | x |  |  |
|  |  | G81.x | Hemiplegia | x |  | x |  |
|  | Subarachnoid Haemorrhage | I60 | Subarachnoid haemorrhage | x | x |  |  |
|  |  | I69.0 | Sequalae of subarachnoid haemorrhage | x | x |  |  |
|  | Other stroke | I67 | Other cerebrovascular diseases | x | x |  |  |
|  |  | I68 | Cerebrovascular disorders in diseases classified elsewhere | x | x |  |  |
|  |  | I69.8 | Sequelae of other and unspecified cerebrovascular diseases | x | x |  |  |

Note : * ICD-10 codes not mapped from the Elixhauser Comorbidity Index: AIDS/HIV , Peptic Ulcer Disease, Pulmonary Circulation disorders, Hypothyroidism, Coagulopathy, Obesity, Weight loss, Fluid and electrolyte disorders, Blood loss anaemia, Deficiency Anaemia, Alcohol abuse, Drug abuse, Psychoses.
